# Editorial of Special Issue “Body Image Perception and Body Composition in Different Populations: The Role of Physical Education and Sport”

**DOI:** 10.3390/ejihpe12110119

**Published:** 2022-11-20

**Authors:** Gianpiero Greco

**Affiliations:** Department of Translational Biomedicine and Neuroscience (DiBraiN), University of Study of Bari, 70124 Bari, Italy; gianpiero.greco@uniba.it

Body image is the dynamic perception of one’s body—how it looks, feels, and moves; it can change with mood, physical experience, and environment [1]. There are many different factors affecting body image, including gender, the media, parental relationships, and puberty, as well as weight and popularity [2]. In Western society, the ideal body for males is muscular and lean, whereas, for females, a thin body is viewed as more desirable. For both genders, the desire to alter shape or weight during adolescence is common. It is associated with emotional distress and dramatic measures to alter appearances such as cosmetic surgery, depression, eating disorders, and exercise addiction [3,4].

Physical education and sports practice result in optimizing body composition and a more positive body image. The benefit of physical education and sport on body image has been demonstrated by previous studies confirming the greater satisfaction of adolescents with their bodies [5,6]. The effects of different physical performance parameters and their relation to body composition features are widely known [7,8]. Additionally, the assessment of body composition over a competitive season could provide valuable information that can help sports professionals to evaluate the efficacy of training and nutritional strategies, as well as monitor athletes’ health status [9]. Body composition assessment is often used for tactical populations as an important tool for the evaluation of physical fitness and health status [10]. Therefore, it is valuable to assess body composition since it is the result of various factors such as diet, stress, physical activity, and other daily habits [11].

This Special Issue aimed to collect and disseminate the most current research examining the role of physical education and sport in the development of positive body image perception through education for a healthy lifestyle and the achievement of optimal body composition.

In the study by Belli et al. [12], the topic of adolescent idiopathic scoliosis was discussed. This condition can cause alterations in psychological components such as self-perceived body image and self-identity, which can negatively affect adolescents’ quality of life. Therefore, through a cross-sectional study design, the authors aimed to investigate how fifteen participants with a diagnosis of mild adolescent idiopathic scoliosis perceived their body image and how the mild magnitude of the curve impacted their quality of life. The findings showed that, although the scoliosis was not severe, the mild magnitude of the spinal curve negatively affects self-perceived body image, as measured by the Scoliosis Research Society Patient Questionnaire in subjects with adolescent idiopathic scoliosis. Finally, the authors recommended that more attention should be paid to the quality of life of adolescents with idiopathic scoliosis and that early treatment may be necessary to prevent psychological disorders related to self-perception.

Stojkovic and colleagues [13] argue that an accurate evaluation of obesity and body composition assessment in Police Trainees is highly important, especially for the early identification and prevention of obesity, in order to be healthier and more productive at work once they become Police Officers. Therefore, in their study, the authors aimed to compare BMI classification as an indirect method of measuring obesity to the direct method of body fat percentage to determine how BMI classifies subjects who possess different levels of skeletal muscle mass percentages. Additionally, they aimed to determine the prevalence of overweight and obesity status among Police Trainees. Their findings indicated that muscular Police Trainees could be misclassified as overweight and that body fat percentage identified more subjects as obese. Namely, three Police Trainees were obese according to BMI, while thirteen were obese according to body fat percentage. The information provided by this research could be used to help professionals understand the importance of measuring body composition and the inaccuracies of BMI classification. This is especially important in the tactical population given that they must always be physically ready to perform sometimes highly demanding professional duties. The authors concluded that, whenever possible, skeletal muscle mass percentage and body fat percentage should replace the utilization of BMI to screen overweight and obesity in Police Trainees. Agencies may consider using BIA as a non-invasive, quick, and inexpensive measurement tool.

In their brief report, Tindaro et al. [14] stated that, from a practical perspective, the phase angle (PhA) represents a simple, quick, and non-invasive value that could be used to monitor the overall fitness and body composition features of soccer players during the competitive season. The assessment of PhA can be considered together with other parameters related to physical performance, such as muscle strength and endurance level, in the search for the players’ optimal physical condition. In addition, since PhA can be measured either in the whole body or in the different hemisomes, it is important to study which measure may be more informative in the evaluation of a soccer player. Therefore, the authors aimed to verify, through a longitudinal pilot study design, the association between changes in the lower hemisoma PhA and vertical jump in elite soccer players. They hypothesized that changes in the lower hemisoma PhA could be more strongly related to changes in vertical jump performance than that concerning the whole-body PhA. The results showed that changes in the lower hemisoma PhA are more strongly related to changes in jump performance than changes in the whole-body PhA, even after adjusting for the legs’ lean soft tissue and body mass index. Their findings suggest that changes in the lower hemisoma PhA might be used as a tool for evaluating performance-related parameters in sports where specific body segments are involved, in preference to the whole-body measured value. Finally, this pilot study, conducted during a competitive Serie A soccer season, suggests that an increase in the lower limb PhA appears to be associated with an increase in vertical jump performance in elite soccer players.

In another pilot study, Tindaro et al. [15] premised that the assessment of body composition over a competitive season provides valuable information that can help sports professionals to evaluate the efficacy of training and nutritional strategies, as well as monitor athletes’ health status. Therefore, they investigated whether changes in body composition and body water were related to lower-body neuromuscular performance changes throughout a competitive season. In soccer, given the high demand imposed on the lower limbs during a season, they hypothesized that regional (i.e., leg) lean soft tissue (LST) would be more susceptible to change in response to the entire competitive season period, as compared to other whole-body parameters (e.g., whole-body LST or fat-free mass). The results showed that changes in leg LST and intracellular water (ICW) significantly explained the improvements in the countermovement jump height, power, and strength over an entire competitive season in professional soccer players. Their findings support that assessing regional body composition would be more informative for monitoring lower-limb neuromuscular changes over the competitive season. Therefore, the current preliminary results encourage practitioners, coaching staff, sports scientists, and sports nutritionists to employ countermovement jump measures while also assessing their athletes by tracking ICW and regional LST to derive quantitative and qualitative information on the training status and inherent characteristics of athletes. For instance, they could help to identify the individual strengths and weaknesses of physical performance (e.g., jumping tasks) while enabling more targeted, appropriate, and realistic training plans.

In summary, this editorial summarized some important studies that have shown how physical and sports education programs could help to improve the body image and quality of life of adolescents. Furthermore, it was discussed that the assessment of body composition is fundamental for the tactical population who must always be ready to perform professional tasks, and for athletes’ performance in competitive sports such as soccer, where the measurement of regional body composition is more informative.
